# Supplementary material for: Single Cell Transcriptomic and Chromatin Profiles Suggest Layer Vb Is the Only Layer With Shared Excitatory Cell Types in the Medial and Lateral Entorhinal Cortex
Source: Front Neural Circuits. 2022 Jan 26;15:806154. doi: 10.3389/fncir.2021.806154 (PMC8826650; doi:10.3389/fncir.2021.806154)
Supplement: Supplementary file 14 [file Data_Sheet_1.ZIP › Inh_vs_Exc_Motifs/homerResults/motif2.similar.html]

motif2

## Information for motif2

C
A
T
G
A
T
C
G
G
A
C
T
C
T
G
A
C
G
T
A
A
G
C
T
A
G
C
T
C
T
G
A
  
Reverse Opposite:  

G
A
C
T
T
C
G
A
C
T
G
A
C
G
A
T
A
G
C
T
C
T
G
A
T
A
G
C
G
T
A
C
  

|  |  |
| --- | --- |
| p-value: | 1e-141 |
| log p-value: | -3.265e+02 |
| Information Content per bp: | 1.647 |
| Number of Target Sequences with motif | 4347.0 |
| Percentage of Target Sequences with motif | 35.51% |
| Number of Background Sequences with motif | 9705.2 |
| Percentage of Background Sequences with motif | 25.16% |
| Average Position of motif in Targets | 281.6 +/- 233.5bp |
| Average Position of motif in Background | 336.4 +/- 294.9bp |
| Strand Bias (log2 ratio + to - strand density) | -0.1 |
| Multiplicity (# of sites on avg that occur together) | 1.34 |
| Motif File: | file (matrix) reverse opposite |

### Similar de novo motifs found

|  |  |  |  |  |  |  |  |
| --- | --- | --- | --- | --- | --- | --- | --- |
| Rank | Match Score | Redundant Motif | P-value | log P-value | % of Targets | % of Background | Motif file |
| 1 | 0.958 | C T A G A T G C G C A T T A C G G A C T T C G A C G T A C G A T A G C T C T G A | 1e-136 | -314.032567 | 43.92% | 33.09% | motif file (matrix) |
| 2 | 0.895 | C A G T A T G C C G A T C T A G C G T A A G C T A C G T C T A G T A G C C G T A C A T G G T A C | 1e-120 | -277.445530 | 25.54% | 17.13% | motif file (matrix) |
| 3 | 0.762 | G A T C C G A T T C A G T C G A G A C T C A G T A C T G A T G C C T A G G A C T | 1e-77 | -177.825763 | 10.44% | 6.03% | motif file (matrix) |
| 4 | 0.608 | T G C A C T G A A C G T A G T C G C T A A C T G T A G C G C T A | 1e-73 | -168.328868 | 30.01% | 22.90% | motif file (matrix) |
| 5 | 0.863 | T A G C G C A T C T G A C G T A C G A T A T G C C T G A T C A G | 1e-71 | -164.252412 | 32.88% | 25.61% | motif file (matrix) |
| 6 | 0.791 | T A G C G C A T T C G A C G T A G C A T A C G T C G T A G A C T | 1e-47 | -108.230212 | 42.70% | 36.34% | motif file (matrix) |
| 7 | 0.825 | G A C T T C G A C G T A G A C T A G C T C G A T T A C G A T C G | 1e-42 | -98.411679 | 55.95% | 49.73% | motif file (matrix) |
| 8 | 0.663 | C G A T T G C A G C T A C G A T A G C T C G T A C T A G G T A C C A T G G A T C G C A T A G T C | 1e-33 | -77.962421 | 29.80% | 24.93% | motif file (matrix) |
| 9 | 0.604 | A G T C A G C T T C A G C G T A C G T A C G A T T C A G T C A G | 1e-32 | -75.868114 | 41.86% | 36.57% | motif file (matrix) |
| 10 | 0.711 | C A T G A G T C A G C T G A C T A T C G T G C A C G A T A G C T T G C A A T C G T G C A A C T G | 1e-29 | -68.346195 | 5.11% | 3.15% | motif file (matrix) |
| 11 | 0.732 | A T G C C G T A G A C T T G A C C T G A G A C T A G C T C G T A | 1e-28 | -65.033374 | 25.19% | 21.01% | motif file (matrix) |
| 12 | 0.680 | G C T A C T G A C G T A A C G T G A C T C T A G G T A C C A T G | 1e-27 | -63.787391 | 25.64% | 21.47% | motif file (matrix) |
| 13 | 0.764 | G T A C G C A T T C G A C G T A C G A T A C G T G C T A C G A T C T A G G A T C G T A C G C A T | 1e-20 | -48.345709 | 35.00% | 30.98% | motif file (matrix) |
| 14 | 0.716 | A T C G T G A C G C T A T C G A C G T A A C G T A T G C C G T A T C A G C A G T | 1e-19 | -43.983191 | 35.67% | 31.83% | motif file (matrix) |
| 15 | 0.605 | A C G T A C G T A C T G C G T A A C G T A C G T A C T G C G T A | 1e-2 | -5.443117 | 2.05% | 1.73% | motif file (matrix) |
| 16 | 0.685 | C G T A C G T A C G T A C G T A A C G T A C G T C G T A A G T C | 1e0 | -1.297672 | 3.33% | 3.23% | motif file (matrix) |
